# Fast X-ray Differential Phase Contrast Imaging with One Exposure and without Movements

**DOI:** 10.1038/s41598-018-37687-0

**Published:** 2019-02-04

**Authors:** Jian Fu, Xianhong Shi, Wei Guo, Peng Peng

**Affiliations:** 1 Research Center of Digital Radiation Imaging and Biomedical Imaging, Beihang University, Beijing, 100191 China; 2 School of Mechanical Engineering and Automation, Beihang University, Beijing, 100191 China

## Abstract

Grating interferometry X-ray differential phase contrast imaging (GI-XDPCI) has provided enhanced imaging contrast and attracted more and more interests. Currently the low imaging efficiency and increased dose remain to be the bottlenecks in the engineering applications of GI-XDPCI. Different from the widely-used X-ray absorption contrast imaging (XACI) found in hospitals and factories, GI-XDPCI involves a grating stepping procedure that is time-consuming and leads to a significantly increased X-ray exposure time. In this paper, we report a fast GI-XDPCI method without movements by designing a new absorption grating. There is no grating stepping in this approach, and all components remain stationary during the imaging. Three kinds of imaging contrasts are provided with greatly reduced time. This work is comprised of a numerical study of the method and its verification using a sub-set of the dataset measured with a standard GI-XDPCI system at the beam line BL13W1 of the Shanghai Synchrotron Radiation Facility (SSRF). These results have validated the presented method.

## Introduction

X-ray imaging has become increasingly popular since its discovery by German scientist Röntgen in 1895. Not only does it help to cure diseases, but it also develops new materials and provides public safety. Today X-ray imaging has been closely related to our lives.

X-ray imaging has long been based on the absorption of X-ray in different materials. This is referred to as X-ray absorption contrast imaging (XACI). Good contrast is achieved by XACI between materials with high-density differences, such as between bones and soft-tissues. Unfortunately, XACI can not make a distinction among soft-tissues since they usually have similar absorption properties. Recently, the phase shift of X-rays passing through matter has been developed as the imaging contrast, and this corresponding method is referred to as X-ray phase contrast imaging (XPCI). This method yields much better imaging contrast compared to XACI for materials that have a low atomic number such as soft-tissues, nonmetallic composites, and insects.

Inspired by the visible light phase contrast microscopy invented by Swiss scientist Zernike in the 1930s, several differential techniques have been proposed over last decades to measure the phase shift of X-rays and form the phase contrast images such as propagation-based^1^, analyzer-based^2,3^, edge illumination^4,5^, speckled tracking^6–8^, far-field interferometry^9^, beam tracking^10,11^, and interferometric effects-based methods^12–14^. Different from other approaches, the last category does not rely on the high brilliant and coherent X-ray source, and has already attracted much interest with it being implemented by conventional X-ray tube sources^14^. It modulates the phase changes into intensity variations by using Talbot or Talbot-Lau grating interferometers and then demodulates the recorded X-ray intensity signal to obtain the phase gradient imaging contrast perpendicular to the gratings, giving the method its other common name: grating interferometry X-ray differential phase contrast imaging (GI-XDPCI). Preliminary experimental results have demonstrated its potential to provide soft-tissue contrast significantly higher than XACI, as well as deliver additional and complementary information^15–22^.

**Figure 1:**
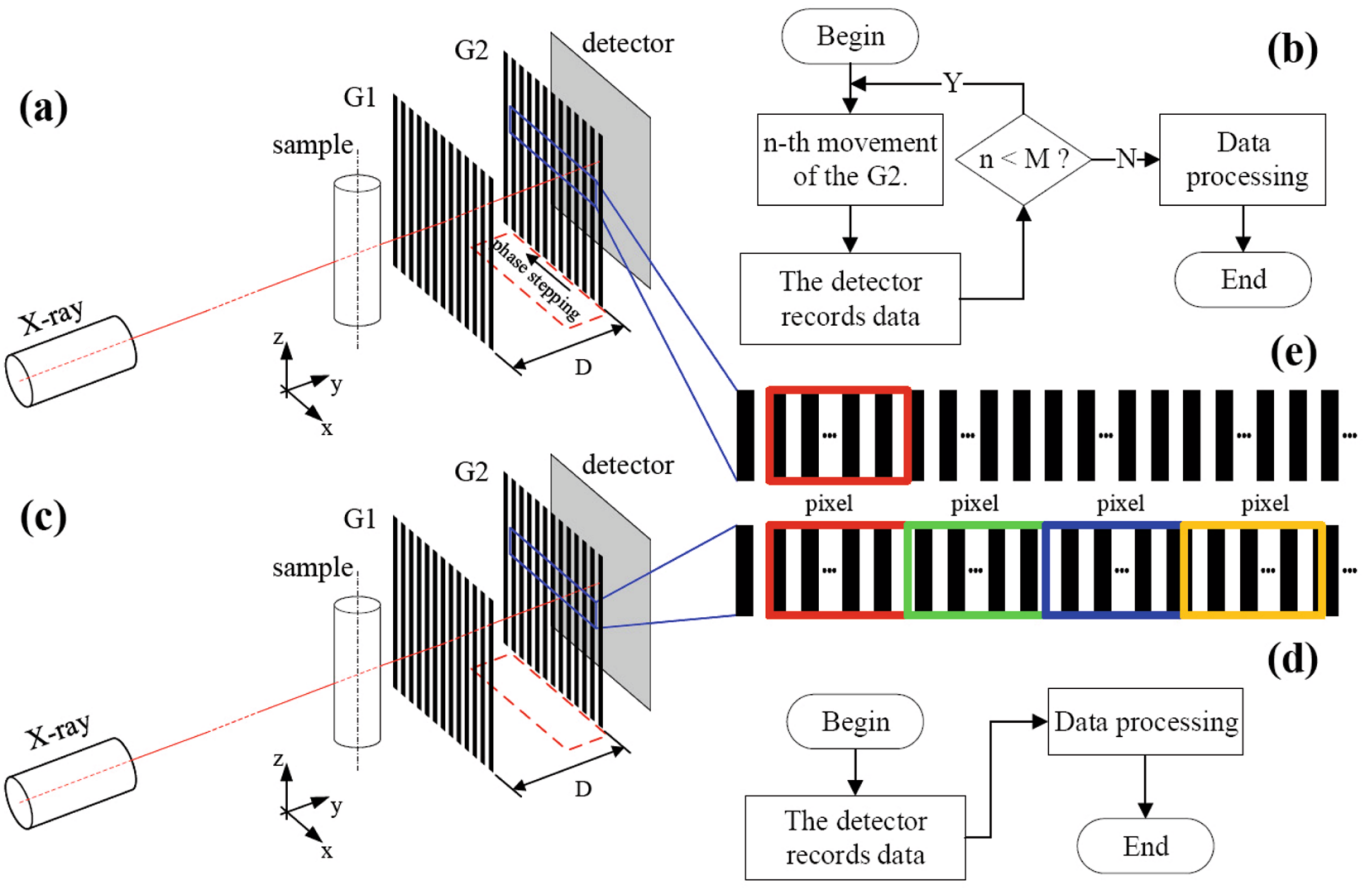
GI-XDPCI configurations. (**a**) is the setup of the conventional method with a phase stepping procedure and (**b**) the phase retrieval steps. (**c**) is the configuration of the proposed method without movements and (**d**) the implementation steps. (**e**) Gives a structure comparison of absorption gratings between the conventional method and the proposed method.

In a typical GI-XDPCI system, such as that depicted in Fig. 1(a), one of the two gratings consisting of interferometer G1 and G2 will translate several times to record the interferometric patterns of the sample at multiple positions. This procedure is called phase stepping (PS), and provides a shifting curve for each detector pixel. By applying the analytical or statistical analysis methods to the shifting curves, a two-dimensional phase contrast image is finally retrieved. Compared with the conventional XACI in which all components remain stationary, GI-XDPCI is time-consuming and leads to an increased dose because of the existence of PS.

Several techniques have recently been proposed to relax PS. Zhu *et al*. adopted a first-order Taylor expansion to linearly approximate the shifting curve near the half slope and achieved a method called reverse projection (RP)^23^. For this, an initialization grating scanning is first executed to determine the center of the linear region of the shifting curve and two interferometric patterns of the sample are then acquired at two opposite angles. The analysis from Wu *et al*. shows that RP has a superior sensitivity and provides refraction images with a higher signal noise ration compared to PS^24^. However, it needs to rotate the sample 180° and uses two projections to retrieve the phase contrast signal. Moreover, the retrieved phase is only correct for small refraction angles, limiting its applications. It is also incapable of obtaining a dark-field signal usually provided by PS. RP - similar to that of the other methods mentioned below - is better suited to phase contrast computed tomography (PCCT) since PCCT needs to intrinsically rotate samples. Based on RP, Wu *et al*. also developed a linear information retrieval method and demonstrated its interchangeability with tomographic reconstruction^25^.

Zanette *et al*. reported an interlaced acquisition scheme in which the phase step and the PCCT rotation step are combined together. Different viewing angles are used to retrieve a single differential phase projection^26^, permitting continuous PCCT rotation scanning and providing a higher accuracy for the region-of-interest PCCT. However, it is still based on PS and grating translation is necessary.

Kottle *et al*. described a fringe-scanning method^27^. Gratings are first misaligned to form vertical or horizontal Moire fringe and then the sample, instead of the gratings, is moved step by step along the direction perpendicular to the Moire fringe. The phase signal is finally obtained from the intensity projection images recorded by the multiple line detectors with precisely defined distances between them. Although it intrinsically belongs to PS, the movement of gratings is replaced by that of samples, significantly improving the stability of the system. This technique has also been extended to helical computed tomography^28^, as well as being implemented at synchrotron facilities and in a laboratory setup^29,30^. Based on this method, Roessl *et al*. developed a commercial mammography system^31^.

Teufenbach *et al*. proposed a single-shot PCCT method^32^ based on a statistical iterative reconstruction (SIR) algorithm and a sliding window acquisition pattern^33^ or the electromagnetic phase-stepping technique^34^. The successful application of these techniques demonstrates that using the right reconstruction approach and a suitable acquisition pattern allows us to eliminate the most critical challenges in grating-based PCCT.

However, it should be noted that movements always exist in the above-mentioned approaches. Moreover, at least two projection images are used to obtain the phase contrast image, leading to an increased exposure time and X-ray dose. The work from Ge *et al*. demonstrated that it is possible to extract phase signal from one exposure^35^. They developed an absorption grating to eliminate the additional data acquisition time needed to perform PS. In this grating, the linear structures are shifted from one detector row to the next and the amount of the lateral shift was equal to a fraction of the X-ray diffraction fringe pattern. The X-ray data from several neighboring detector rows were then combined to extract differential phase information. Initial experimental results have demonstrated that it enables accurate phase signal acquisition from a single X-ray exposure. An equivalent design has also been presented using the edge illumination method^36,37^.

In this work, we present an alternative design of absorption gratings to implement phase retrieval with only one projection image, while simultaneously allowing all components - including gratings, detectors, sources and samples - to remain stationary.

Figure 1(a) depicts the configuration of a standard GI-XDPCI setup. Phase grating G1 and absorption grating G2 consist of Talbot interferometer. Their periods and intervals are elaborately designed to match the corresponding Talbot interference effect. When X-rays penetrate samples (going through G1 and G2 and reaching the detector behind G2), the intensity pattern image, or the projection image, is formed and recorded. Next, G2 is shifted along the x-direction M times (typically M = 3–16) and the corresponding projections are acquired at these positions. This procedure is referred to as phase stepping and briefly shown in Fig. 1(b). As an example, M is set to be four in this article.

Similar to that of the classical setup in Fig. 1(a,b), the sketch and the imaging steps of the designed GI-XDPCI system are given in Fig. 1(c,d). The difference lies within the new absorption grating G2 - it is the most iconic design and different from the conventional one. Shown in Fig. 1(e), the linear structures are shifted from one detector column to the next in this new absorption grating G2. It is fabricated in a way that it intrinsically features different phase stepping positions for different detector regions. With one exposure, the intensity signals from several adjacent pixels in the recorded projection image can be used to form the shifting curve of the current pixel and retrieve the corresponding phase signal. The entire procedure is presented in Fig. 1(d). There is clearly no movement in this implementation and the sample is exposed only once. Compared to the conventional method, the total number of exposures is reduced by a factor of M-1. As such, it has the potential to reduce exposure time, increase efficiency, and improve system stability. The new grating G2 and the data processing will be discussed in the Methods section.

## Results

In this section, we present the numerical and experimental results of GI-XDPCI with only one exposure and without movements by using the proposed method.

### Numerical results

**Figure 2:**
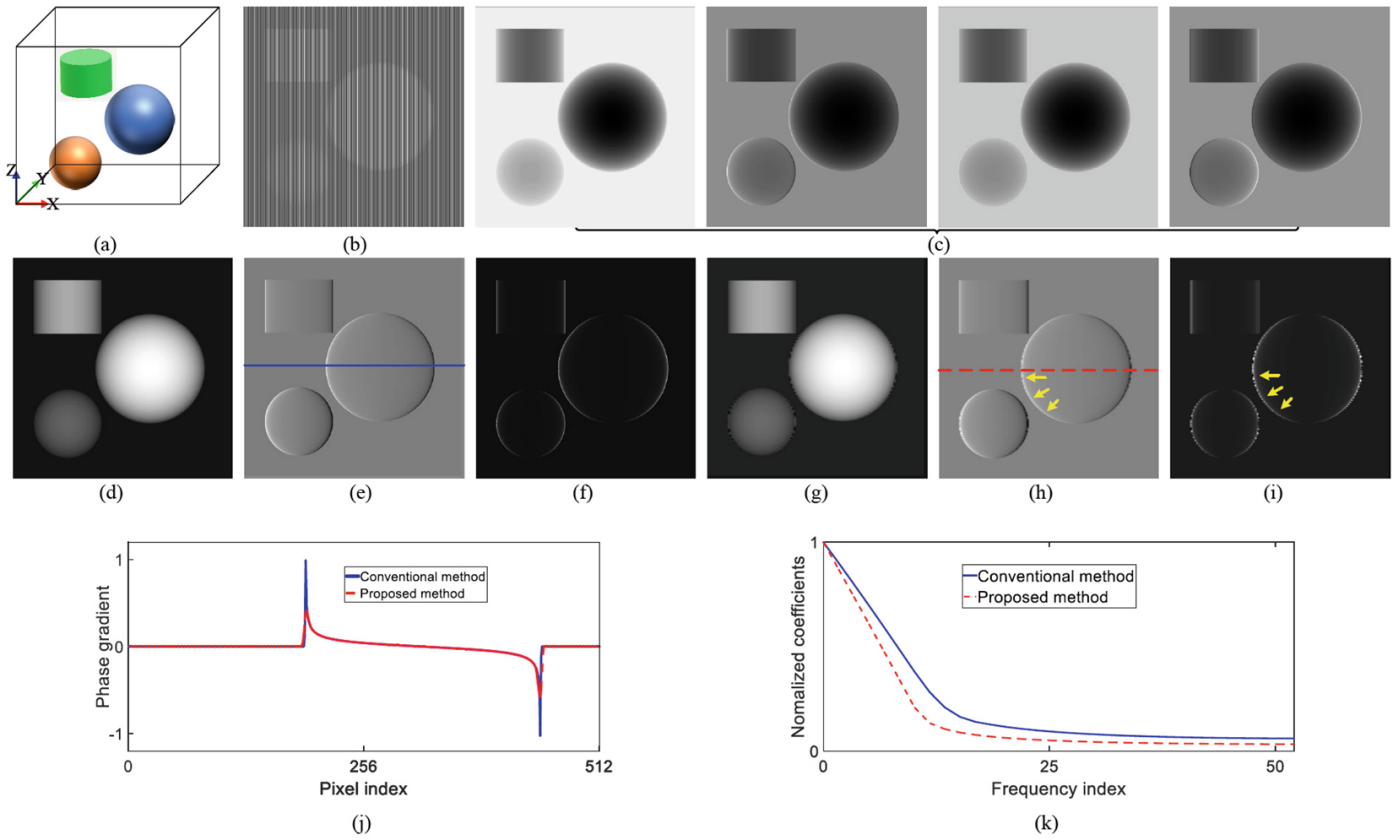
Numerical simulation results. (**a**) is the phantom. (**b**) is the original projection image recorded by the proposed method. (**c**) Shows the four original projection images from the conventional PS procedure. (**d**–**f**) are the absorption, phase, and dark-field contrast images retrieved by the conventional PS, respectively. (**g**–**i**) The absorption, phase, and dark-field contrast images retrieved by the proposed method, respectively. (**j**) Presents the grey value curves along the blue line in (**e**) and the red line in (**h**). (**k**) Shows the Fourier transform curves of the two curves in (**j**).

As shown in Fig. 2(a), a numerical simulation was executed to validate the proposed method with a phantom consisting of two spheres and one cylinder. Figure 2(b) shows the original intensity projection image recorded by the proposed method. The four original intensity projection images recorded by PS are shown in Fig. 2(c). Here, the number of steps in PS is set to be four. Figure 2(d,g) are, respectively, the retrieved absorption contrast images by PS and the new method. Figure 2(e,h) are the differential phase contrast images. Figure 2(f,i) are the dark field images. Figure 2(j) presents the grey value curves along the red and blue lines in Fig. 2(e,h). Comparing the results in Fig. 2(b,c), it can be observed that there are some vertical stripe patterns contained in the projection image of the proposed method and non-existed in the ones of PS. This is caused by the new absorption grating in which the linear structures are shifted from one detector column to the next and the amount of the shift is equal to a fraction of the X-ray diffraction fringe pattern. In this example, the amount is four to match the number of phase steps in PS.

Furthermore, the result in Fig. 2(e) seems to coincide with the one in Fig. 2(h), qualitatively demonstrating that the proposed method can retrieve out the phase gradient signal with a contrast comparable to that of PS except that some edge-blurring occurs in the horizontal direction. In order to quantitatively evaluate the results, the correlation coefficient of Fig. 2(e,h) was calculated. The value of this parameter generally lies within the range of 0 to 1. The bigger the number is, the more they match. The calculated value is 0.9052, indicating that these two figures match well. The correlation coefficient of the two curves in Fig. 2(j) was also calculated. The value is 0.9688 and again supports the above conclusion.

The edge-blurring is attributable to the fact that the neighbouring detector pixels are combined to retrieve the phase signal in the proposed method. The Fourier transform curves - obtained by applying Fourier transform to the curves in Fig. 2(j) and drawn in Fig. 2(k) - show that the new method indeed has a lower modulation value. At high frequencies, the modulation value of the new method is approximately 70% of that of the conventional PS. Even so, the resulted benefits are quite attractive: such as only one exposure, no movements, and high efficiency. In this simulation, in total four projection images are acquired with grating translations in PS. In contrast, only one projection image is recorded without any movement in the new method.

### Experimental results

**Figure 3:**
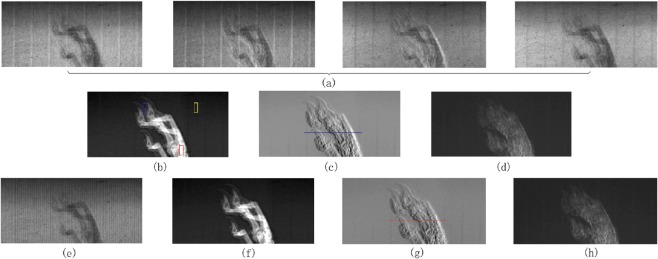
Experimental results. (**a**) Displays the four original projection images recorded by detectors in conventional PS. (**b**–**d**) are the retrieved absorption, phase gradient, and dark-field images from the projections in (a), respectively. (**e**) is the equivalent projection recorded in the proposed method. (**f**–**h**) are the retrieved absorption, phase gradient, and dark-field images from the projection in (**e**), respectively.

An imaging experiment with a hamster front toe was first conducted by PS using a Talbot interferometer. The number of phase steps is four and a total of four projection images of the sample at four grating positions were recorded with an exposure time of 1.5 s per projection. These images are presented in Fig. 3(a). Absorption, differential phase, and dark-field contrast images were then extracted by applying Fourier analytical algorithm to these four projections and shown in Fig. 3(b–d), respectively. There are some regular vertical bands from the grating structure in these images.

The new absorption grating is currently unavailable in our experiments. As such, we rearranged some data from the above PS experiment to act as an equivalent projection of the new method, as shown in Fig. 3(e). It is a sub-set of the experimental dataset acquired by PS. This rearrangement operation is described in the Methods section. As anticipated, the vertical stripe patterns appear in this rearranged equivalent original projection image. These kinds of patterns are unique in the new method and their appearance validates this rearrangement operation. Following absorption, phase, and dark-field contrast images were retrieved by using the same Fourier analytical algorithm with this equivalent projection. These are shown in Fig. 3(f–h), respectively.

By comparing the results in Fig. 3(b–d,f–h), one can observe that the new method enables the retrieval of three imaging contrast signals with only one exposure and without any movement. The downside is that the new method has the increased noise and reduced resolution along the horizontal direction.

**Figure 4:**
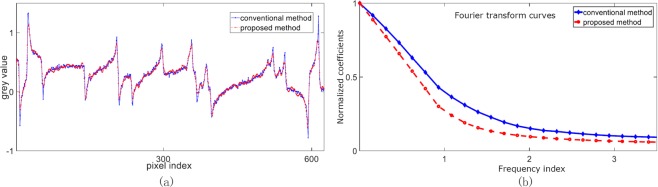
Spatial resolution analysis results. (**a**) Displays the grey value curves drawn along the red line in Fig. 3(c) and the blue line in Fig. 3(g). (**b**) Presents the Fourier transform curves of the proposed and conventional methods.

In order to evaluate the spatial resolution of the phase contrast images in Fig. 3(c,g) in a quantitative manner, Fourier transform was applied to the grey value curves in Fig. 4(a), which are drawn along the blue line in Fig. 3(c) and the red line in Fig. 3(g). Figure 4(b) presents the corresponding Fourier transform curves. At high frequencies, the modulation value of the new method is about 65% of that of the conventional PS. This reduction is caused by the combination of neighboring pixels in the proposed method.

## Discussion

The new method does not involve any movement such as grating translation, sample rotation, and helical motion. Moreover, only one exposure is needed to retrieve the imaging signal. Consequently, it is faster than PS and also it is helpful in improving the stability of the imaging system. The exposure time is also reduced by a factor (M-1). Here, M is the number of the phase steps in PS.

**Figure 5:**
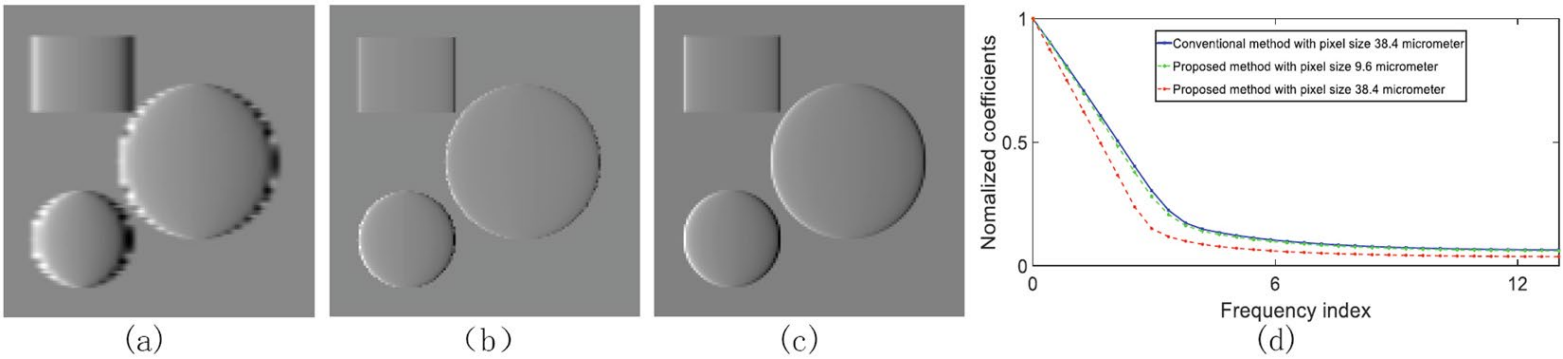
Spatial resolution analysis with different detector pixel size. (**a**,**b**) are the phase contrast images by the new method with pixel sizes 38.4 *μ*m and 9.6 *μ*m, respectively. (**c**) is the result by the conventional PS with pixel size of 38.4 *μ*m. (**d**) Displays their respective Fourier transform curves.

The reduction on spatial resolution is the side effect. This can be improved in the future by using an advanced detector with better detective quantum efficiency (DQE) and smaller pixel size, or by developing better image processing algorithms. Although a thorough discussion is beyond the scope of this article, a preliminary numerical analysis was still executed as an example to demonstrate that it is possible to improve the spatial resolution by adopting a detector with smaller pixel size. In this analysis, the above numerical simulation was repeated with pixel size of 38.4 *μ*m. Figure 5(a,b) are the phase contrast images by the new method with pixel sizes 38.4 *μ*m and 9.6 *μ*m, respectively. Figure 5(c) is the result by the conventional PS with pixel size of 38.4 *μ*m. Figure 5(d) displays their respective Fourier transform curves. At any frequency, the modulation value with pixel size of 9.6 *μ*m is always better than the one with pixel size of 38.4 *μ*m for the new method. Moreover, the modulation value of the new method with pixel size of 9.6 *μ*m is almost the same as the ones of the conventional PS with pixel size of 38.4 *μ*m, simply demonstrating that the new method can improve the spatial resolution by using advanced detector with smaller pixel size. Of course, limited by the working principle, the new method always has a lower resolution than PS when they adopt the same detector.

Image artifact is another side effect. In the proposed method, the values recorded by several consecutive pixels are used to retrieve three imaging signals, leading to image artifacts since each one of these consecutive pixels corresponds to a different portion of the sample. The situation is similar to the one in the technique with vertical staggered absorption gratings^35^. It adopted several vertically consecutive pixels to retrieve the imaging signals. On the contrary, these pixels in the proposed method are consecutive horizontally. In principle, both cannot accurately extract the information along the vertical or horizontal direction when attenuation and refraction vary on a scale that is smaller than these pixels. For the proposed method, it will lead to edge distortion and sawtooth artifacts along the horizontal direction. Indicated by the yellow arrows in Fig. 2(h,i), they are perfectly demonstrated along the sphere circumference in the phase contrast and dark field images retrieved by the new method. The sphere phantom is quite suitable to visualize this problem since the tangential slope around the circumference continuously varies from zero to infinity. Noticeably, the larger the slope becomes, the more serious the artifacts seem to be.

**Figure 6:**
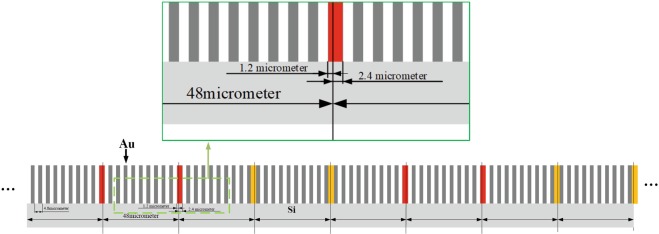
The fabrication design of the new absorption grating with LIGA processing. Silicon wafers work as substrates. The gold bars occlude X-rays. Each element of this grating has a size of 48 *μ*m × 48 *μ*m to match the pixel size of a CMOS X-ray flat panel detector. The grating has a period of 4.8 *μ*m and so each element contains 10 gold bars.

The new absorption grating plays a key role in the realization of the proposed method. It will be fabricated with LIGA technique by Microworks GmbH, a spin-off from the Institute of Microstructure Technology (IMT) at the Karlsruhe Institute of Technology (KIT) in Germany. Figure 6 shows the fabrication design of the new absorption grating. Since Silicon wafer has a low absorption of hard X-rays, it is chosen as the substrate of the grating. The bars are made of gold with LIGA processing and occlude X-rays. Each element of this grating has a size of 48 *μ*m × 48 *μ*m to match the pixel size of a CMOS X-ray flat panel detector (Shad-o-Box^*TM*^ 2048, Rad-icon Imaging Corp., California, USA). The grating has a period of 4.8 *μ*m and so each element contains 10 gold bars. It has an active area 4.9 cm × 4.9 cm and work with a *π*-phase Talbot X-ray diffraction grating designed to operate at a mean X-ray energy of 28 keV and have a pitch of 8 *μ*m.

## Methods

### New absorption grating

**Figure 7:**
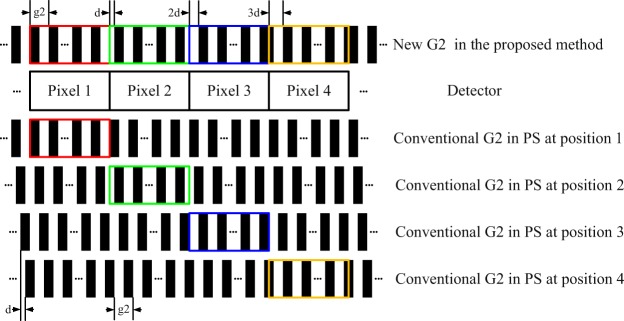
The structure of the new absorption grating.

The idea of the new absorption grating is that the projections recorded by multiple adjacent pixels in a row of the detector can be considered as the ones of a pixel at multiple phase stepping positions in conventional PS. Accordingly, only one single-shot image is recorded to retrieve phase contrast signal supported by the new grating. In order to implement this idea, it is specially designed to contain many groups of Si/Au linear structures. Each group is interposed with an offset distance *d* and the linear structures have a period g2 within a group. The width of each group equals the size of a detector pixel. *d* and g2 have a relationship depicted in Eq. (1). Here *M* is the number of phase stepping in PS. As an example, the new grating with an *M* of four is shown in Fig. 7. Four colored boxes indicate four groups of linear structures in the new grating corresponding to the ones in PS.

1 $$d=\frac{1}{M}$$

### Signal retrieval

One can use Fourier analysis or statistical algorithms to retrieve the imaging contrast signals. The former, depicted in Eqs (2–4) maintains a good balance between image quality and efficiency and was applied to the extracted dataset to obtain three kinds of imaging contrast signals. Here *a*_0_ and *a*_1_ are the zero-order and first-order coefficients of the Fourier transform of the shifting curve. *ϕ*_1_ is the phase of the first-order component. Finally, three images of the sample, absorption A, phase P, and dark-field D, were formed by using the operations in Eqs (5–7), in which the superscripts *r* and *s* represent “reference” and “sample”, respectively. Here, *I* is the projection image obtained in the new method, while *X* and *Z* are, respectively, the row and column indexes of detector pixels.

2 $${a}_{0}(X,Z)=\frac{1}{M}\sum _{m=1}^{M}I(X+3-m,Z)$$

3 $${a}_{1}(X,Z)=\frac{2}{M}\sqrt{{[{\sum }_{m=1}^{M}I(X+3-m,Z)\sin (\frac{2\pi m}{M})]}^{2}+{[{\sum }_{m=1}^{M}I(X+3-m,Z)\cos (\frac{2\pi m}{M})]}^{2}}$$

4 $${\varphi }_{1}(X,Z)=ta{n}^{-1}[-\frac{{\sum }_{m=1}^{M}I(X+3-m,Z)\,\sin (\frac{2\pi m}{M})}{{\sum }_{m=1}^{M}I(X+3-m,Z)\,\cos (\frac{2\pi m}{M})}]$$

5 $$A(X,Z)=\frac{{a}_{0}^{s}}{{a}_{0}^{r}}$$

6 $$P(X,Z)={\varphi }_{1}^{s}-{\varphi }_{1}^{r}$$

7 $$D(X,Z)=A(X,Z)=\frac{{a}_{0}^{r}}{{a}_{0}^{s}}\frac{{a}_{1}^{s}}{{a}_{1}^{r}}$$

### Numerical simulation setup

The simulation is based on the well-established Huygens-Fresnel principle stating that at any given moment a wavefront may be considered as the sum of spherical wavelets distributed on the wavefront. A 50% duty ideal phase grating with a period of 2.4 *μm* periodically shifts the phase of the incoming wave by *π*/2. An ideal absorption grating with a period of 2.4 *μm* with 50% duty is placed 4.66 cm behind of the phase grating. The detector with a pixel size of 9.6 *μm* is right behind of the absorption grating. The captured image size is 512 × 512 pixels.

The used 3D phantom consists of a polypropylene (PP) sphere with a diameter of 2.56 mm, a carbon sphere with a diameter of 1.6 mm, and a polymethyl methacrylate (PMMA) cylinder with a diameter of 1.6 mm and a height of 1.26 mm. The selected X-ray wavelength is 0.061 nm and corresponds to 20.080 keV. The values of *β* and *δ* of the PP sphere are set to be 1.6036 *e*^−10^ and 5.2867 *e*^−7^, for the carbon sphere 3.0290 *e*^−10^ and 8.7475 *e*^−7^ and for the PMMA cylinder 2.8198 *e*^−10^ and 6.6122 *e*^−7^ respectively.

### Experimental setup

The experiments were conducted using a Talbot grating at the beam line BL13W1 of the Shanghai Synchrotron Radiation Facility (SSRF). The phase grating G1 was made of Ni with a period 2.396 *μ*m and shifts the phase of the incoming wave *π*/2. The absorption grating G2 was made of Au with a period 2.4 *μ*m. The distance between G1 and G2 was set to be 4.64 cm and corresponds to the 1st Talbot distance. A CCD camera with a pixel size of 9 *μ*m was placed 16 cm behind the absorption grating G2. The used X-ray energy was 20 keV.

The sample is a hamster front toe and four projection images were collected at equally spaced positions over one period of the shifting curve. The exposure time was 1.5 s for each projection image.

### Data rearrangement

Eqs (8–11) present the experimental data rearrangement. In these equations, *I* is the projection image obtained in the new method. *I*_1_, *I*_2_, *I*_3_, and *I*_4_ represent the projection images by the detector at four phase stepping position in PS, respectively. *X* and *Z* are the row and column indexes of detector pixels.

8 $$I(X-1,Z)={I}_{1}(X,Z)$$

9 $$I(X,Z)={I}_{2}(X,Z)$$

10 $$I(X+1,Z)={I}_{3}(X,Z)$$

11 $$I(X+2,Z)={I}_{4}(X,Z)$$
